# The Development and Implementation of a Parallel, Multi-Campus Medical Anatomy Curriculum Delivered with and without Cadavers

**DOI:** 10.1007/s40670-024-02179-6

**Published:** 2024-09-25

**Authors:** William Harvey, Lachlan Van Schaik, Hayden Frizzell, Julian Wright, Michelle M. Rank

**Affiliations:** 1 Department of Rural Health, The University of Melbourne, 49 Graham Street, Shepparton, VIC Australia; 2 Department of Anatomy and Physiology, The University of Melbourne, Melbourne, VIC Australia

**Keywords:** Interactive multimedia, Practical learning sessions, 3D models, Rural

## Abstract

A parallel, multi-campus anatomy curriculum was developed that could be delivered with or without body donor cadaveric teaching resources. This blended program includes asynchronous lectures, e-Learning modules, formative self-assessment, independent study, and synchronous hands-on practical sessions. Practical sessions at the metropolitan campus utilise professionally prepared body donor cadaveric teaching resources, whereas rural campus practical sessions use a combination of non-cadaveric resources such as 3D models (physical and digital) and interactive multimedia to achieve identical learning outcomes. Here, we discuss the specific features of the practical sessions delivered in this novel cross-campus curriculum, with a focus on the non-cadaveric teaching resources deployed.

## Background

Anatomy has a long history of face-to-face, hands-on teaching that traditionally relies heavily on didactic-style teaching alongside dissection and professionally prepared human cadaveric specimens (prosections) [1]. Although these methods remain a worthy cornerstone of the discipline, recent innovations in alternative methods such as digital and physical 3D models and interactive multimedia have been increasingly incorporated into modern medical training programs. Such methods have been employed to good effect, without disadvantaging students in their assessment results [2]. These alternative modalities offer increased flexibility for learners, greater support in self-directed learning, and increased ease in accessibility as the resources are available to students for longer periods and over a wider variety of times. These modern resources have the benefit of being effective alternatives to traditional cadaveric resources used in practicals and, importantly, are cost-effective solutions for institutions that lack appropriate infrastructure or access to human body donor programs and ethically derived cadaveric teaching resources.

The University of Melbourne developed an end-to-end rural Doctor of Medicine Course, situated in Shepparton, a regional city with an estimated population of less than 70,000 [3], with the first cohort of post-graduate medical students inducted in 2022. Importantly, the curriculum content for the Doctor of Medicine course was required to be identical for both rural (~ 30 students) and metropolitan (~ 300 students) programs. The metropolitan campus is located in Melbourne, a capital city with an estimated population of over 4.9 million [4].

Given the necessity to deliver identical anatomical curricula to both rural and metropolitan cohorts, a key question in setting up the new rural anatomy education pathway was not *what* to teach, but instead *how* to teach it. A well-resourced Human Body Donor program is administered on the metropolitan campus; therefore, a robust collection of high-quality human cadaveric teaching resources are available for use in hands-on anatomy practical classes. Conversely, the rural Shepparton campus does not have access to human cadaveric teaching resources. The resources for hands-on practical classes were therefore a central consideration in designing the curriculum delivery for the rural pathway program.

Here, we describe the practical anatomy sessions that were developed for first year medical students (MD1) on the rural campus. These sessions do not depend on the use of cadaveric specimens and are run in parallel with the cadaveric specimen-based anatomy practicals delivered on the metropolitan campus. We deployed specialised interactive multimedia resources (*Complete Anatomy* and *Sectra IDS7*), 3D models (digital resources and physical models), surface anatomy, clinical anatomy, and medical imaging.

## Activity

### Overview of Practical Sessions

**Fig. 1:**
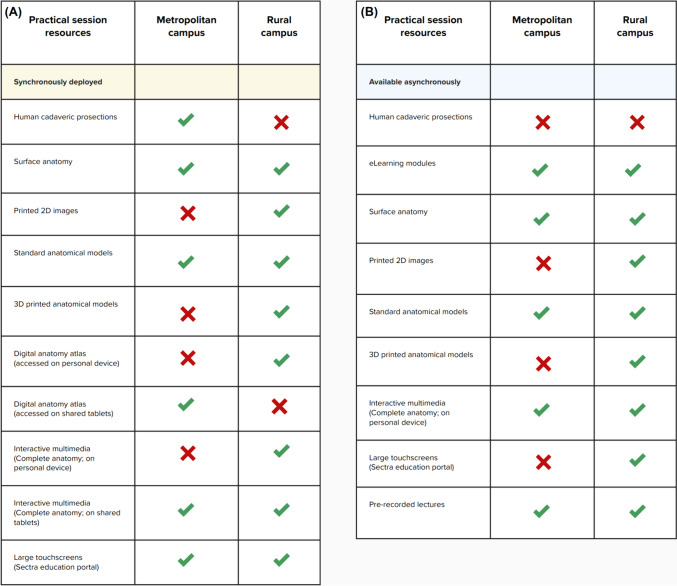
A pair of tables comparing the **A** synchronously deployed and **B** asynchronously deployed components in the rural and/or metropolitan first-year post-graduate medical degree anatomy curriculum

The anatomical learning in the MD1 curriculum delivers identical learning outcomes for cohorts located on metropolitan and rural campuses. Seven practical sessions are conducted in MD1, each focusing on a specific area of regional anatomy (i.e. thorax, abdomen, upper limb, lower limb, head, neck, and pelvis + perineum). For each practical session, accompanying asynchronous resources as described below and outlined in Fig. 1 (i.e. pre-recorded lectures, eLearning modules, self-assessment quizzes) are made available to both metropolitan and rural student cohorts with the expectation that the asynchronous content is completed prior to attendance at synchronous hands-on practical session.

Synchronous hands-on practical sessions are 3 h in duration and featured a station-based design with five discrete stations each focussing on a specific topic area (i.e. osteology, joints, muscles, nerves/vessels, and medical imaging). A dedicated station based around medical imaging is incorporated into each practical, where students interact with radiological images. Equity of access to imaging resources at rural and metropolitan campuses is ensured via the purchase of large touchscreens (*Sectra IDS7*) with dedicated 3D reconstruction and radiological imaging suites via *Sectra Education Portal* licences.

### Design Elements Specific to Practical Sessions at the Rural Campus

The first curriculum element developed was a bespoke suite of e-Learning modules utilising *Articulate Rise*. These consist of text-based learning, high-definition cadaveric photographs, 3D digital anatomical scans of cadaveric prosections, diagrammatic images, flash cards, clinical scenarios, and applied anatomy formative assessment. e-Learning modules were deliberately designed to complement the hands-on practical sessions. Importantly, the 3D scans in the e-Learning modules are of the same human cadaveric prosections utilised in the synchronous hands-on practical sessions on the metropolitan campus. This essential asynchronous resource ensures that all students, rural and metropolitan, can study human anatomy independently in an authentic context outside of limited formal practical lab experiences.

For synchronous hands-on practical sessions on the rural campus, a combination of 3D models (both digital and physical), hands-on surface anatomy activities, interactive multimedia (including *Complete Anatomy* app and *Sectra IDS7* medical imaging resources), and 2D image-based resources (i.e. anatomical atlases) are used. Resources were carefully selected to ensure anatomical variation, relations, and scale are represented and could be integrated into the students’ learning experience. Unique 3D-printed models are also used; these models are based on scans of human body donors and thus are to scale and representative of authentic human anatomy.

## Discussion

Unique and bespoke alternative learning resources, which did not rely on human cadaveric specimens, are utilised for anatomy practicals in a first-year post-graduate medical degree in a rural setting. These practical sessions address identical content and learning objectives to anatomy practicals delivered in a metropolitan setting that uses human cadaveric resources as a central learning tool. There are several points to consider when designing hands-on practicals that use non-cadaveric anatomy teaching resources. Scale, anatomical variation, and anatomical relations are critical features of the learning resources that were selected for use.

A unique benefit when deploying interactive multimedia, 3D models (both digital and physical), and surface anatomy for hands-on practical sessions is the ability for students to access these resources outside of designated class times. The deliberate design of practical sessions around these flexible resources allows students to revisit, revise, or even recreate practical sessions independently. This is an important aspect of facilitating the development of self-directed adult learners and is a particularly important proficiency for medical students to develop [5]. In our experience, students on the rural campus approach their anatomy learning by utilising interactive multimedia on personal devices most often, followed by surface anatomy, and then physical 3D models. We feel that our model of a parallel curriculum could be readily deployed at other multi-campus institutions to provide an equitable learning experience where human cadaveric specimens are not viable at all campuses.

Central to our parallel curriculum is the use of interactive multimedia resources in place of human cadaveric specimens. Such resources are a valuable and effective vector for delivering and contextualising anatomy content. Interactive multimedia is often based on medical imaging (i.e. CT and MRI scans and 3D-rendered CT scans) that present real and varied anatomy to students. The authentic nature of these images may stimulate interest and meaning in students not engaged by idealised artworks or 3D physical models [6]. Importantly, it is these modalities of medical imaging that students will be regularly exposed to in the clinical setting.

Consideration must be paid to the limitations of the use of physical and digital non-cadaveric anatomy resources. While there are some pre-developed session packages on multimedia platforms such as the *Sectra Education Portal*, these sessions are unlikely to perfectly align with the curriculum of various institutions and degrees. As such, the time-intensive task of developing bespoke sessions on these interactive multimedia platforms is required and is the approach favoured by our team. A consideration in the use of such digital platforms is the training and familiarisation period for associated staff, which can require many dozens of hours of upskilling and knowledge uplift for teaching staff. Procurement of physical 3D anatomy models also requires careful consideration as manufacturers and suppliers often have long lead-times (up to 9–12 months). Therefore, specific models must be selected and ordered well ahead of scheduled class delivery.

Regardless of the resources used to teach anatomy, either cadaveric or other supplementary physical and digital teaching and learning resources, we concur with the following five points considered essential for effective and engaging anatomy teaching in a medical course [7]: (1) learning must be student centred, (2) content must be integrated wherever possible, (3) clinically applicable scenarios should be incorporated wherever possible, (4) student learning must be continually observed and analysed, and (5) the outcomes of new educational interventions must be assessed, and these findings must be published in the literature.

Here, we have described the development of a parallel, multi-campus anatomy curriculum across metropolitan and rural campuses, with and without access to human cadaveric resources, respectively. We ensured that the curriculum is student-centred, integrated, clinically applicable and utilises a wide variety of learning resources. Ongoing work will evaluate this educational intervention and compare learning and engagement outcomes across rural and metropolitan cohorts in the medical program.
